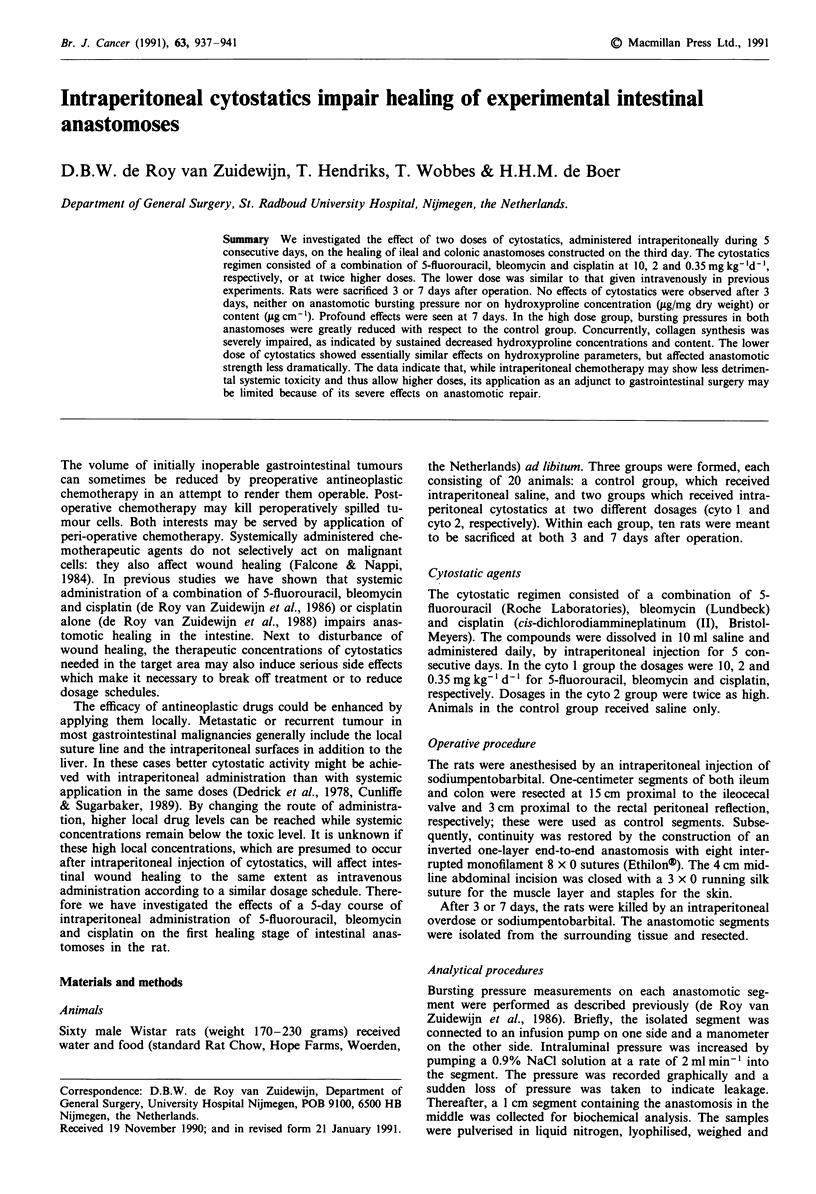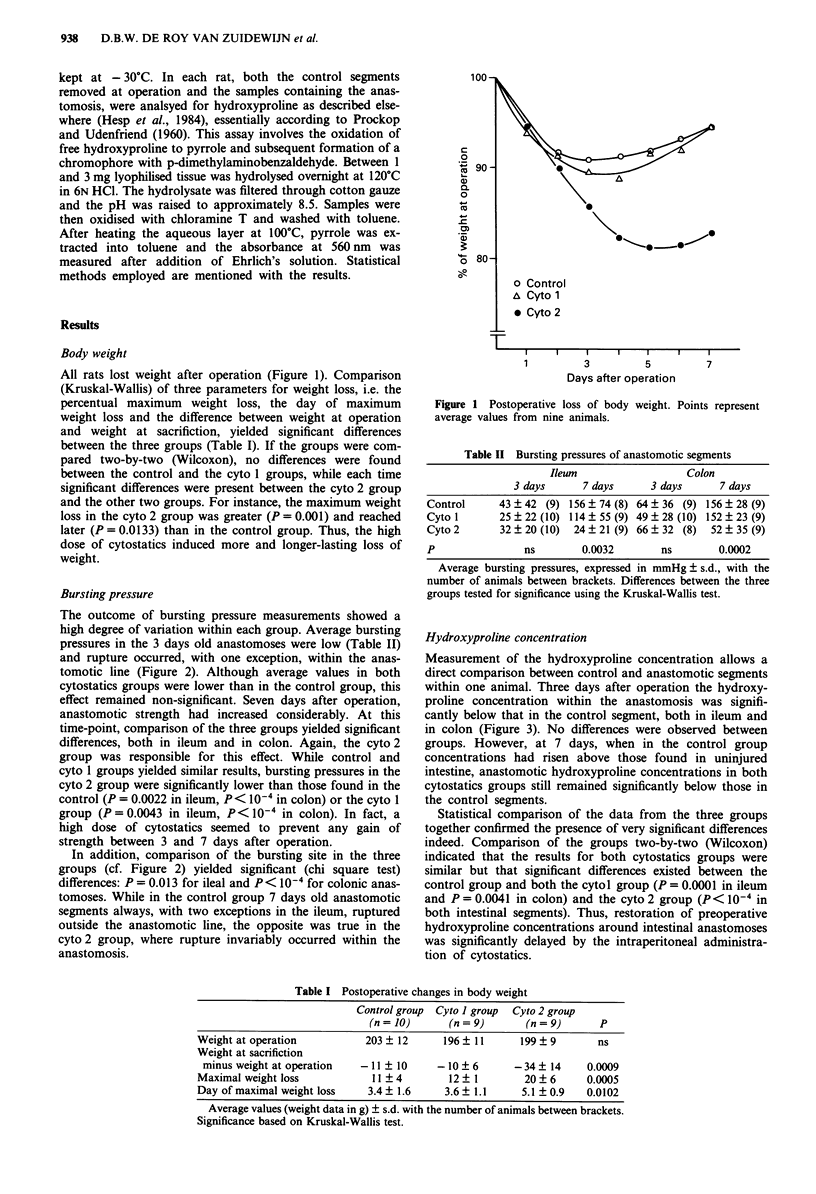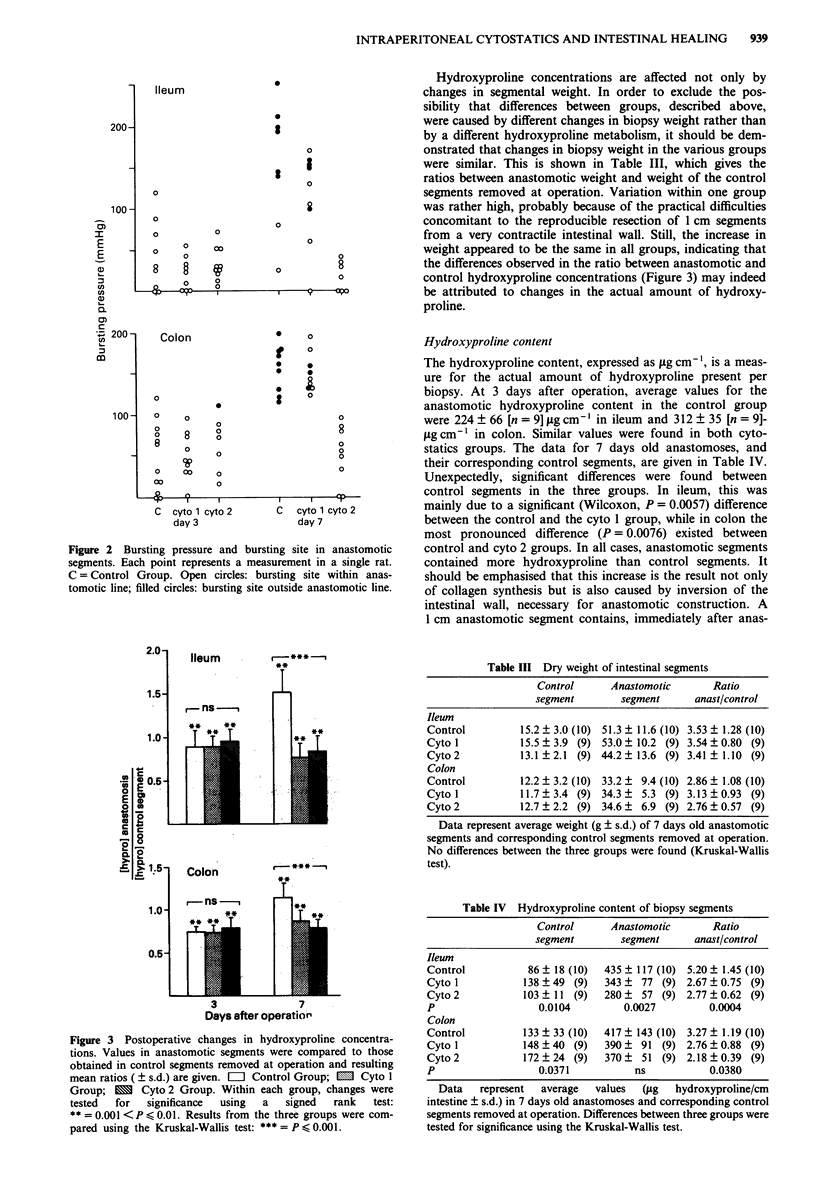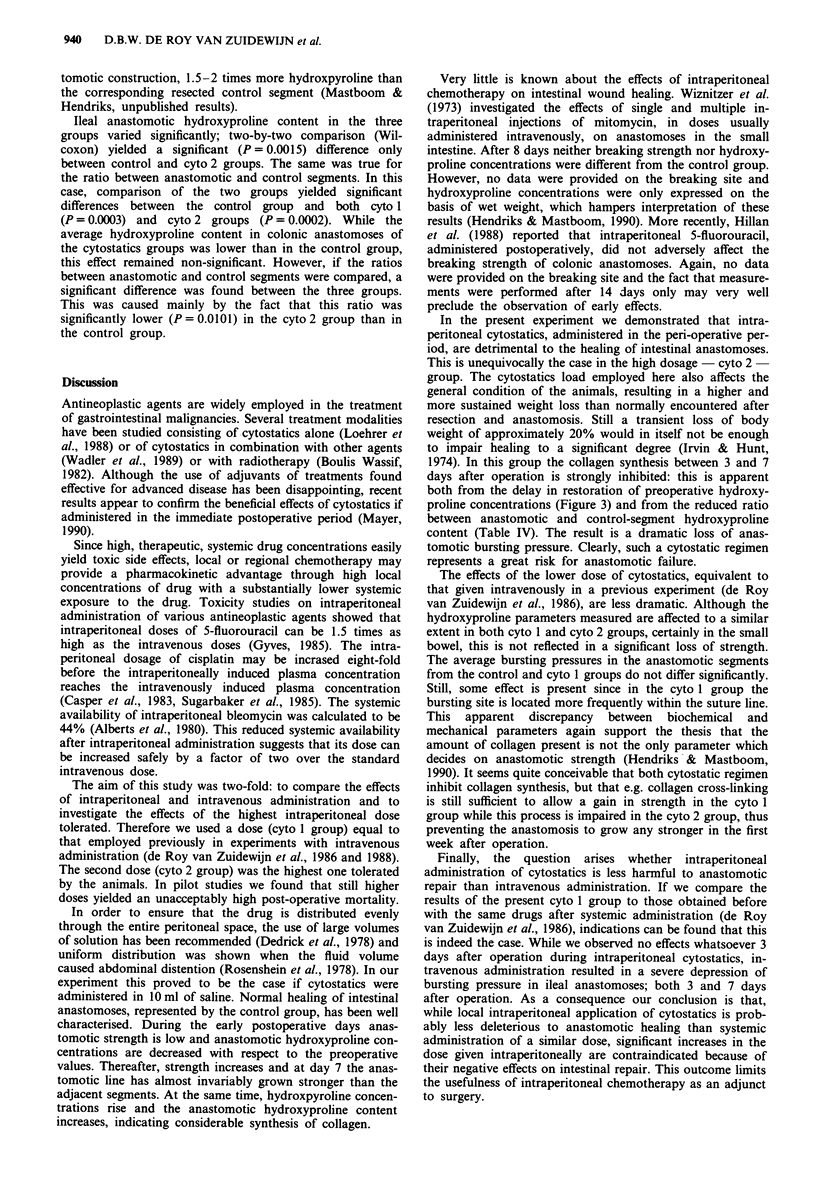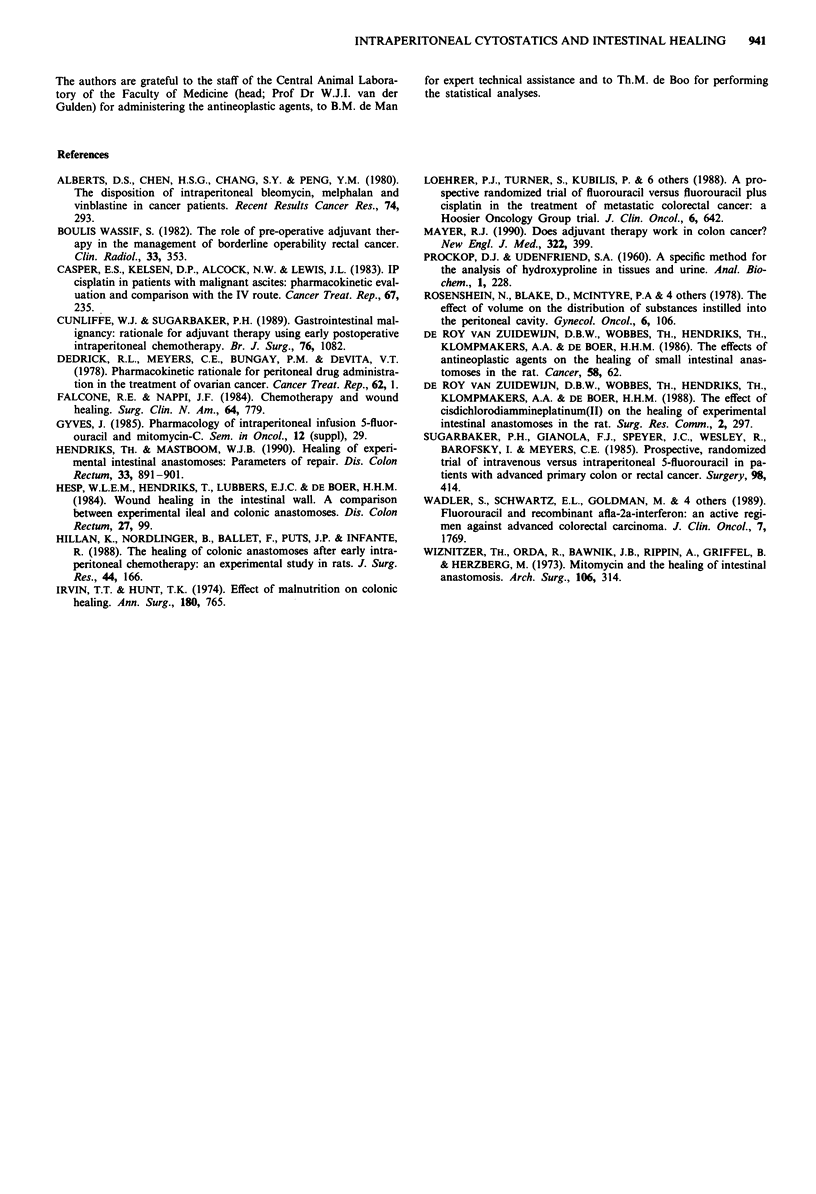# Intraperitoneal cytostatics impair healing of experimental intestinal anastomoses.

**DOI:** 10.1038/bjc.1991.205

**Published:** 1991-06

**Authors:** D. B. de Roy van Zuidewijn, T. Hendriks, T. Wobbes, H. H. de Boer

**Affiliations:** Department of General Surgery, St. Radboud University Hospital, Nijmegen, The Netherlands.

## Abstract

We investigated the effect of two doses of cytostatics, administered intraperitoneally during 5 consecutive days, on the healing of ileal and colonic anastomoses constructed on the third day. The cytostatics regimen consisted of a combination of 5-fluorouracil, bleomycin and cisplatin at 10, 2 and 0.35 mg kg-1d-1, respectively, or at twice higher doses. The lower dose was similar to that given intravenously in previous experiments. Rats were sacrificed 3 or 7 days after operation. No effects of cytostatics were observed after 3 days, neither on anastomotic bursting pressure nor on hydroxyproline concentration (microgram/mg dry weight) or content (microgram cm-1). Profound effects were seen at 7 days. In the high dose group, bursting pressures in both anastomoses were greatly reduced with respect to the control group. Concurrently, collagen synthesis was severely impaired, as indicated by sustained decreased hydroxyproline concentrations and content. The lower dose of cytostatics showed essentially similar effects on hydroxyproline parameters, but affected anastomotic strength less dramatically. The data indicate that, while intraperitoneal chemotherapy may show less detrimental systemic toxicity and thus allow higher doses, its application as an adjunct to gastrointestinal surgery may be limited because of its severe effects on anastomotic repair.


Br. J. Cancer (1991), 63, 937-941 © Macmillan Press Ltd., 1991

### Intraperitoneal cytostatics impair healing of experimental intestinal anastomoses

D.B.W. de Roy van Zuidewijn, T. Hendriks, T. Wobbes & H.H.M. de Boer

*Department of General Surgery, St. Radboud University Hospital, Nijmegen, the Netherlands.*

**Summary** We investigated the effect of two doses of cytostatics, administered intraperitoneally during 5 consecutive days, on the healing of ileal and colonic anastomoses constructed on the third day. The cytostatics regimen consisted of a combination of 5-fluorouracil, bleomycin and cisplatin at 10, 2 and 0.35 mg $kg^{-1}d^{-1}$, respectively, or at twice higher doses. The lower dose was similar to that given intravenously in previous experiments. Rats were sacrificed 3 or 7 days after operation. No effects of cytostatics were observed after 3 days, neither on anastomotic bursting pressure nor on hydroxyproline concentration (μg/mg dry weight) or content (μg $cm^{-1}$). Profound effects were seen at 7 days. In the high dose group, bursting pressures in both anastomoses were greatly reduced with respect to the control group. Concurrently, collagen synthesis was severely impaired, as indicated by sustained decreased hydroxyproline concentrations and content. The lower dose of cytostatics showed essentially similar effects on hydroxyproline parameters, but affected anastomotic strength less dramatically. The data indicate that, while intraperitoneal chemotherapy may show less detrimental systemic toxicity and thus allow higher doses, its application as an adjunct to gastrointestinal surgery may be limited because of its severe effects on anastomotic repair.

The volume of initially inoperable gastrointestinal tumours can sometimes be reduced by preoperative antineoplastic chemotherapy in an attempt to render them operable. Postoperative chemotherapy may kill peroperatively spilled tumour cells. Both interests may be served by application of peri-operative chemotherapy. Systemically administered chemotherapeutic agents do not selectively act on malignant cells: they also affect wound healing (Falcone & Nappi, 1984). In previous studies we have shown that systemic administration of a combination of 5-fluorouracil, bleomycin and cisplatin (de Roy van Zuidewijn *et al.*, 1986) or cisplatin alone (de Roy van Zuidewijn *et al.*, 1988) impairs anastomotic healing in the intestine. Next to disturbance of wound healing, the therapeutic concentrations of cytostatics needed in the target area may also induce serious side effects which make it necessary to break off treatment or to reduce dosage schedules.

The efficacy of antineoplastic drugs could be enhanced by applying them locally. Metastatic or recurrent tumour in most gastrointestinal malignancies generally include the local suture line and the intraperitoneal surfaces in addition to the liver. In these cases better cytostatic activity might be achieved with intraperitoneal administration than with systemic application in the same doses (Dedrick *et al.*, 1978, Cunliffe & Sugarbaker, 1989). By changing the route of administration, higher local drug levels can be reached while systemic concentrations remain below the toxic level. It is unknown if these high local concentrations, which are presumed to occur after intraperitoneal injection of cytostatics, will affect intestinal wound healing to the same extent as intravenous administration according to a similar dosage schedule. Therefore we have investigated the effects of a 5-day course of intraperitoneal administration of 5-fluorouracil, bleomycin and cisplatin on the first healing stage of intestinal anastomoses in the rat.

### Materials and methods

### *Animals*

Sixty male Wistar rats (weight 170–230 grams) received water and food (standard Rat Chow, Hope Farms, Woerden, the Netherlands) *ad libitum*. Three groups were formed, each consisting of 20 animals: a control group, which received intraperitoneal saline, and two groups which received intraperitoneal cytostatics at two different dosages (cyto 1 and cyto 2, respectively). Within each group, ten rats were meant to be sacrificed at both 3 and 7 days after operation.

### *Cytostatic agents*

The cytostatic regimen consisted of a combination of 5-fluorouracil (Roche Laboratories), bleomycin (Lundbeck) and cisplatin (*cis*-dichlorodiammineplatinum (II), Bristol-Meyers). The compounds were dissolved in 10 ml saline and administered daily, by intraperitoneal injection for 5 consecutive days. In the cyto 1 group the dosages were 10, 2 and 0.35 mg $kg^{-1}$ $d^{-1}$ for 5-fluorouracil, bleomycin and cisplatin, respectively. Dosages in the cyto 2 group were twice as high. Animals in the control group received saline only.

### *Operative procedure*

The rats were anesthesised by an intraperitoneal injection of sodiumpentobarbital. One-centimeter segments of both ileum and colon were resected at 15 cm proximal to the ileocecal valve and 3 cm proximal to the rectal peritoneal reflection, respectively; these were used as control segments. Subsequently, continuity was restored by the construction of an inverted one-layer end-to-end anastomosis with eight interrupted monofilament 8 × 0 sutures (Ethilon®). The 4 cm midline abdominal incision was closed with a 3 × 0 running silk suture for the muscle layer and staples for the skin.

After 3 or 7 days, the rats were killed by an intraperitoneal overdose or sodiumpentobarbital. The anastomotic segments were isolated from the surrounding tissue and resected.

### *Analytical procedures*

Bursting pressure measurements on each anastomotic segment were performed as described previously (de Roy van Zuidewijn *et al.*, 1986). Briefly, the isolated segment was connected to an infusion pump on one side and a manometer on the other side. Intraluminal pressure was increased by pumping a 0.9% NaCl solution at a rate of 2 ml $min^{-1}$ into the segment. The pressure was recorded graphically and a sudden loss of pressure was taken to indicate leakage. Thereafter, a 1 cm segment containing the anastomosis in the middle was collected for biochemical analysis. The samples were pulverised in liquid nitrogen, lyophilised, weighed and

Correspondence: D.B.W. de Roy van Zuidewijn, Department of General Surgery, University Hospital Nijmegen, POB 9100, 6500 HB Nijmegen, the Netherlands.
Received 19 November 1990; and in revised form 21 January 1991.

kept at −30°C. In each rat, both the control segments removed at operation and the samples containing the anastomosis, were analsyed for hydroxyproline as described elsewhere (Hesp *et al.*, 1984), essentially according to Prockop and Udenfriend (1960). This assay involves the oxidation of free hydroxyproline to pyrrole and subsequent formation of a chromophore with p-dimethylaminobenzaldehyde. Between 1 and 3 mg lyophilised tissue was hydrolysed overnight at 120°C in 6N HCl. The hydrolysate was filtered through cotton gauze and the pH was raised to approximately 8.5. Samples were then oxidised with chloramine T and washed with toluene. After heating the aqueous layer at 100°C, pyrrole was extracted into toluene and the absorbance at 560 nm was measured after addition of Ehrlich's solution. Statistical methods employed are mentioned with the results.

### Results

### *Body weight*

All rats lost weight after operation (Figure 1). Comparison (Kruskal-Wallis) of three parameters for weight loss, i.e. the percentual maximum weight loss, the day of maximum weight loss and the difference between weight at operation and weight at sacrifiction, yielded significant differences between the three groups (Table I). If the groups were compared two-by-two (Wilcoxon), no differences were found between the control and the cyto 1 groups, while each time significant differences were present between the cyto 2 group and the other two groups. For instance, the maximum weight loss in the cyto 2 group was greater ($P = 0.001$) and reached later ($P = 0.0133$) than in the control group. Thus, the high dose of cytostatics induced more and longer-lasting loss of weight.

### *Bursting pressure*

The outcome of bursting pressure measurements showed a high degree of variation within each group. Average bursting pressures in the 3 days old anastomoses were low (Table II) and rupture occurred, with one exception, within the anastomotic line (Figure 2). Although average values in both cytostatics groups were lower than in the control group, this effect remained non-significant. Seven days after operation, anastomotic strength had increased considerably. At this time-point, comparison of the three groups yielded significant differences, both in ileum and in colon. Again, the cyto 2 group was responsible for this effect. While control and cyto 1 groups yielded similar results, bursting pressures in the cyto 2 group were significantly lower than those found in the control ($P = 0.0022$ in ileum, $P < 10^{-4}$ in colon) or the cyto 1 group ($P = 0.0043$ in ileum, $P < 10^{-4}$ in colon). In fact, a high dose of cytostatics seemed to prevent any gain of strength between 3 and 7 days after operation.

In addition, comparison of the bursting site in the three groups (cf. Figure 2) yielded significant (chi square test) differences: $P = 0.013$ for ileal and $P < 10^{-4}$ for colonic anastomoses. While in the control group 7 days old anastomotic segments always, with two exceptions in the ileum, ruptured outside the anastomotic line, the opposite was true in the cyto 2 group, where rupture invariably occurred within the anastomosis.

**Figure 1** Postoperative loss of body weight. Points represent average values from nine animals.

**Table II** Bursting pressures of anastomotic segments

| | Ileum | | Colon | |
|---|---|---|---|---|
| | *3 days* | *7 days* | *3 days* | *7 days* |
| Control | 43 ± 42 (9) | 156 ± 74 (8) | 64 ± 36 (9) | 156 ± 28 (9) |
| Cyto 1 | 25 ± 22 (10) | 114 ± 55 (9) | 49 ± 28 (10) | 152 ± 23 (9) |
| Cyto 2 | 32 ± 20 (10) | 24 ± 21 (9) | 66 ± 32 (8) | 52 ± 35 (9) |
| *P* | ns | 0.0032 | ns | 0.0002 |

Average bursting pressures, expressed in mmHg ± s.d., with the number of animals between brackets. Differences between the three groups tested for significance using the Kruskal-Wallis test.

### *Hydroxyproline concentration*

Measurement of the hydroxyproline concentration allows a direct comparison between control and anastomotic segments within one animal. Three days after operation the hydroxyproline concentration within the anastomosis was significantly below that in the control segment, both in ileum and in colon (Figure 3). No differences were observed between groups. However, at 7 days, when in the control group concentrations had risen above those found in uninjured intestine, anastomotic hydroxyproline concentrations in both cytostatics groups still remained significantly below those in the control segments.

Statistical comparison of the data from the three groups together confirmed the presence of very significant differences indeed. Comparison of the groups two-by-two (Wilcoxon) indicated that the results for both cytostatics groups were similar but that significant differences existed between the control group and both the cytol group ($P = 0.0001$ in ileum and $P = 0.0041$ in colon) and the cyto 2 group ($P < 10^{-4}$ in both intestinal segments). Thus, restoration of preoperative hydroxyproline concentrations around intestinal anastomoses was significantly delayed by the intraperitoneal administration of cytostatics.

**Table I** Postoperative changes in body weight

| | *Control group (n = 10)* | *Cyto 1 group (n = 9)* | *Cyto 2 group (n = 9)* | *P* |
|---|---|---|---|---|
| Weight at operation | 203 ± 12 | 196 ± 11 | 199 ± 9 | ns |
| Weight at sacrifiction minus weight at operation | −11 ± 10 | −10 ± 6 | −34 ± 14 | 0.0009 |
| Maximal weight loss | 11 ± 4 | 12 ± 1 | 20 ± 6 | 0.0005 |
| Day of maximal weight loss | 3.4 ± 1.6 | 3.6 ± 1.1 | 5.1 ± 0.9 | 0.0102 |

Average values (weight data in g) ± s.d. with the number of animals between brackets. Significance based on Kruskal-Wallis test.

**Figure 2** Bursting pressure and bursting site in anastomotic segments. Each point represents a measurement in a single rat. C = Control Group. Open circles: bursting site within anastomotic line; filled circles: bursting site outside anastomotic line.

**Figure 3** Postoperative changes in hydroxyproline concentrations. Values in anastomotic segments were compared to those obtained in control segments removed at operation and resulting mean ratios ( ± s.d.) are given. ☐ Control Group; ▩ Cyto 1 Group; ▧ Cyto 2 Group. Within each group, changes were tested for significance using a signed rank test: ** = $0.001 < P \leq 0.01$. Results from the three groups were compared using the Kruskal-Wallis test: *** = $P \leq 0.001$.

Hydroxyproline concentrations are affected not only by changes in segmental weight. In order to exclude the possibility that differences between groups, described above, were caused by different changes in biopsy weight rather than by a different hydroxyproline metabolism, it should be demonstrated that changes in biopsy weight in the various groups were similar. This is shown in Table III, which gives the ratios between anastomotic weight and weight of the control segments removed at operation. Variation within one group was rather high, probably because of the practical difficulties concomitant to the reproducible resection of 1 cm segments from a very contractile intestinal wall. Still, the increase in weight appeared to be the same in all groups, indicating that the differences observed in the ratio between anastomotic and control hydroxyproline concentrations (Figure 3) may indeed be attributed to changes in the actual amount of hydroxyproline.

### *Hydroxyproline content*

The hydroxyproline content, expressed as $\mu g\ cm^{-1}$, is a measure for the actual amount of hydroxyproline present per biopsy. At 3 days after operation, average values for the anastomotic hydroxyproline content in the control group were 224 ± 66 [$n = 9$] $\mu g\ cm^{-1}$ in ileum and 312 ± 35 [$n = 9$]-$\mu g\ cm^{-1}$ in colon. Similar values were found in both cytostatics groups. The data for 7 days old anastomoses, and their corresponding control segments, are given in Table IV. Unexpectedly, significant differences were found between control segments in the three groups. In ileum, this was mainly due to a significant (Wilcoxon, $P = 0.0057$) difference between the control and the cyto 1 group, while in colon the most pronounced difference ($P = 0.0076$) existed between control and cyto 2 groups. In all cases, anastomotic segments contained more hydroxyproline than control segments. It should be emphasised that this increase is the result not only of collagen synthesis but is also caused by inversion of the intestinal wall, necessary for anastomotic construction. A 1 cm anastomotic segment contains, immediately after anas-

**Table III** Dry weight of intestinal segments

| | *Control segment* | *Anastomotic segment* | *Ratio anast/control* |
|---|---|---|---|
| *Ileum* | | | |
| Control | 15.2 ± 3.0 (10) | 51.3 ± 11.6 (10) | 3.53 ± 1.28 (10) |
| Cyto 1 | 15.5 ± 3.9 (9) | 53.0 ± 10.2 (9) | 3.54 ± 0.80 (9) |
| Cyto 2 | 13.1 ± 2.1 (9) | 44.2 ± 13.6 (9) | 3.41 ± 1.10 (9) |
| *Colon* | | | |
| Control | 12.2 ± 3.2 (10) | 33.2 ± 9.4 (10) | 2.86 ± 1.08 (10) |
| Cyto 1 | 11.7 ± 3.4 (9) | 34.3 ± 5.3 (9) | 3.13 ± 0.93 (9) |
| Cyto 2 | 12.7 ± 2.2 (9) | 34.6 ± 6.9 (9) | 2.76 ± 0.57 (9) |

Data represent average weight (g ± s.d.) of 7 days old anastomotic segments and corresponding control segments removed at operation. No differences between the three groups were found (Kruskal-Wallis test).

**Table IV** Hydroxyproline content of biopsy segments

| | *Control segment* | *Anastomotic segment* | *Ratio anast/control* |
|---|---|---|---|
| *Ileum* | | | |
| Control | 86 ± 18 (10) | 435 ± 117 (10) | 5.20 ± 1.45 (10) |
| Cyto 1 | 138 ± 49 (9) | 343 ± 77 (9) | 2.67 ± 0.75 (9) |
| Cyto 2 | 103 ± 11 (9) | 280 ± 57 (9) | 2.77 ± 0.62 (9) |
| *P* | 0.0104 | 0.0027 | 0.0004 |
| *Colon* | | | |
| Control | 133 ± 33 (10) | 417 ± 143 (10) | 3.27 ± 1.19 (10) |
| Cyto 1 | 148 ± 40 (9) | 390 ± 91 (9) | 2.76 ± 0.88 (9) |
| Cyto 2 | 172 ± 24 (9) | 370 ± 51 (9) | 2.18 ± 0.39 (9) |
| *P* | 0.0371 | ns | 0.0380 |

Data represent average values ($\mu g$ hydroxyproline/cm intestine ± s.d.) in 7 days old anastomoses and corresponding control segments removed at operation. Differences between three groups were tested for significance using the Kruskal-Wallis test.

tomotic construction, 1.5–2 times more hydroxpyroline than the corresponding resected control segment (Mastboom & Hendriks, unpublished results).

Ileal anastomotic hydroxyproline content in the three groups varied significantly; two-by-two comparison (Wilcoxon) yielded a significant ($P = 0.0015$) difference only between control and cyto 2 groups. The same was true for the ratio between anastomotic and control segments. In this case, comparison of the two groups yielded significant differences between the control group and both cyto 1 ($P = 0.0003$) and cyto 2 groups ($P = 0.0002$). While the average hydroxyproline content in colonic anastomoses of the cytostatics groups was lower than in the control group, this effect remained non-significant. However, if the ratios between anastomotic and control segments were compared, a significant difference was found between the three groups. This was caused mainly by the fact that this ratio was significantly lower ($P = 0.0101$) in the cyto 2 group than in the control group.

### Discussion

Antineoplastic agents are widely employed in the treatment of gastrointestinal malignancies. Several treatment modalities have been studied consisting of cytostatics alone (Loehrer *et al.*, 1988) or of cytostatics in combination with other agents (Wadler *et al.*, 1989) or with radiotherapy (Boulis Wassif, 1982). Although the use of adjuvants of treatments found effective for advanced disease has been disappointing, recent results appear to confirm the beneficial effects of cytostatics if administered in the immediate postoperative period (Mayer, 1990).

Since high, therapeutic, systemic drug concentrations easily yield toxic side effects, local or regional chemotherapy may provide a pharmacokinetic advantage through high local concentrations of drug with a substantially lower systemic exposure to the drug. Toxicity studies on intraperitoneal administration of various antineoplastic agents showed that intraperitoneal doses of 5-fluorouracil can be 1.5 times as high as the intravenous doses (Gyves, 1985). The intraperitoneal dosage of cisplatin may be incrased eight-fold before the intraperitoneally induced plasma concentration reaches the intravenously induced plasma concentration (Casper *et al.*, 1983, Sugarbaker *et al.*, 1985). The systemic availability of intraperitoneal bleomycin was calculated to be 44% (Alberts *et al.*, 1980). This reduced systemic availability after intraperitoneal administration suggests that its dose can be increased safely by a factor of two over the standard intravenous dose.

The aim of this study was two-fold: to compare the effects of intraperitoneal and intravenous administration and to investigate the effects of the highest intraperitoneal dose tolerated. Therefore we used a dose (cyto 1 group) equal to that employed previously in experiments with intravenous administration (de Roy van Zuidewijn *et al.*, 1986 and 1988). The second dose (cyto 2 group) was the highest one tolerated by the animals. In pilot studies we found that still higher doses yielded an unacceptably high post-operative mortality.

In order to ensure that the drug is distributed evenly through the entire peritoneal space, the use of large volumes of solution has been recommended (Dedrick *et al.*, 1978) and uniform distribution was shown when the fluid volume caused abdominal distention (Rosenshein *et al.*, 1978). In our experiment this proved to be the case if cytostatics were administered in 10 ml of saline. Normal healing of intestinal anastomoses, represented by the control group, has been well characterised. During the early postoperative days anastomotic strength is low and anastomotic hydroxyproline concentrations are decreased with respect to the preoperative values. Thereafter, strength increases and at day 7 the anastomotic line has almost invariably grown stronger than the adjacent segments. At the same time, hydroxpyroline concentrations rise and the anastomotic hydroxyproline content increases, indicating considerable synthesis of collagen.

Very little is known about the effects of intraperitoneal chemotherapy on intestinal wound healing. Wiznitzer *et al.* (1973) investigated the effects of single and multiple intraperitoneal injections of mitomycin, in doses usually administered intravenously, on anastomoses in the small intestine. After 8 days neither breaking strength nor hydroxyproline concentrations were different from the control group. However, no data were provided on the breaking site and hydroxyproline concentrations were only expressed on the basis of wet weight, which hampers interpretation of these results (Hendriks & Mastboom, 1990). More recently, Hillan *et al.* (1988) reported that intraperitoneal 5-fluorouracil, administered postoperatively, did not adversely affect the breaking strength of colonic anastomoses. Again, no data were provided on the breaking site and the fact that measurements were performed after 14 days only may very well preclude the observation of early effects.

In the present experiment we demonstrated that intraperitoneal cytostatics, administered in the peri-operative period, are detrimental to the healing of intestinal anastomoses. This is unequivocally the case in the high dosage — cyto 2 — group. The cytostatics load employed here also affects the general condition of the animals, resulting in a higher and more sustained weight loss than normally encountered after resection and anastomosis. Still a transient loss of body weight of approximately 20% would in itself not be enough to impair healing to a significant degree (Irvin & Hunt, 1974). In this group the collagen synthesis between 3 and 7 days after operation is strongly inhibited: this is apparent both from the delay in restoration of preoperative hydroxyproline concentrations (Figure 3) and from the reduced ratio between anastomotic and control-segment hydroxyproline content (Table IV). The result is a dramatic loss of anastomotic bursting pressure. Clearly, such a cytostatic regimen represents a great risk for anastomotic failure.

The effects of the lower dose of cytostatics, equivalent to that given intravenously in a previous experiment (de Roy van Zuidewijn *et al.*, 1986), are less dramatic. Although the hydroxyproline parameters measured are affected to a similar extent in both cyto 1 and cyto 2 groups, certainly in the small bowel, this is not reflected in a significant loss of strength. The average bursting pressures in the anastomotic segments from the control and cyto 1 groups do not differ significantly. Still, some effect is present since in the cyto 1 group the bursting site is located more frequently within the suture line. This apparent discrepancy between biochemical and mechanical parameters again support the thesis that the amount of collagen present is not the only parameter which decides on anastomotic strength (Hendriks & Mastboom, 1990). It seems quite conceivable that both cytostatic regimen inhibit collagen synthesis, but that e.g. collagen cross-linking is still sufficient to allow a gain in strength in the cyto 1 group while this process is impaired in the cyto 2 group, thus preventing the anastomosis to grow any stronger in the first week after operation.

Finally, the question arises whether intraperitoneal administration of cytostatics is less harmful to anastomotic repair than intravenous administration. If we compare the results of the present cyto 1 group to those obtained before with the same drugs after systemic administration (de Roy van Zuidewijn *et al.*, 1986), indications can be found that this is indeed the case. While we observed no effects whatsoever 3 days after operation during intraperitoneal cytostatics, intravenous administration resulted in a severe depression of bursting pressure in ileal anastomoses; both 3 and 7 days after operation. As a consequence our conclusion is that, while local intraperitoneal application of cytostatics is probably less deleterious to anastomotic healing than systemic administration of a similar dose, significant increases in the dose given intraperitoneally are contraindicated because of their negative effects on intestinal repair. This outcome limits the usefulness of intraperitoneal chemotherapy as an adjunct to surgery.

The authors are grateful to the staff of the Central Animal Laboratory of the Faculty of Medicine (head; Prof Dr W.J.I. van der Gulden) for administering the antineoplastic agents, to B.M. de Man for expert technical assistance and to Th.M. de Boo for performing the statistical analyses.

### References

ALBERTS, D.S., CHEN, H.S.G., CHANG, S.Y. & PENG, Y.M. (1980). The disposition of intraperitoneal bleomycin, melphalan and vinblastine in cancer patients. *Recent Results Cancer Res.*, **74**, 293.

BOULIS WASSIF, S. (1982). The role of pre-operative adjuvant therapy in the management of borderline operability rectal cancer. *Clin. Radiol.*, **33**, 353.

CASPER, E.S., KELSEN, D.P., ALCOCK, N.W. & LEWIS, J.L. (1983). IP cisplatin in patients with malignant ascites: pharmacokinetic evaluation and comparison with the IV route. *Cancer Treat. Rep.*, **67**, 235.

CUNLIFFE, W.J. & SUGARBAKER, P.H. (1989). Gastrointestinal malignancy: rationale for adjuvant therapy using early postoperative intraperitoneal chemotherapy. *Br. J. Surg.*, **76**, 1082.

DEDRICK, R.L., MEYERS, C.E., BUNGAY, P.M. & DEVITA, V.T. (1978). Pharmacokinetic rationale for peritoneal drug administration in the treatment of ovarian cancer. *Cancer Treat. Rep.*, **62**, 1.

FALCONE, R.E. & NAPPI, J.F. (1984). Chemotherapy and wound healing. *Surg. Clin. N. Am.*, **64**, 779.

GYVES, J. (1985). Pharmacology of intraperitoneal infusion 5-fluorouracil and mitomycin-C. *Sem. in Oncol.*, **12** (suppl), 29.

HENDRIKS, TH. & MASTBOOM, W.J.B. (1990). Healing of experimental intestinal anastomoses: Parameters of repair. *Dis. Colon Rectum*, **33**, 891-901.

HESP, W.L.E.M., HENDRIKS, T., LUBBERS, E.J.C. & DE BOER, H.H.M. (1984). Wound healing in the intestinal wall. A comparison between experimental ileal and colonic anastomoses. *Dis. Colon Rectum*, **27**, 99.

HILLAN, K., NORDLINGER, B., BALLET, F., PUTS, J.P. & INFANTE, R. (1988). The healing of colonic anastomoses after early intraperitoneal chemotherapy: an experimental study in rats. *J. Surg. Res.*, **44**, 166.

IRVIN, T.T. & HUNT, T.K. (1974). Effect of malnutrition on colonic healing. *Ann. Surg.*, **180**, 765.

LOEHRER, P.J., TURNER, S., KUBILIS, P. & 6 others (1988). A prospective randomized trial of fluorouracil versus fluorouracil plus cisplatin in the treatment of metastatic colorectal cancer: a Hoosier Oncology Group trial. *J. Clin. Oncol.*, **6**, 642.

MAYER, R.J. (1990). Does adjuvant therapy work in colon cancer? *New Engl. J. Med.*, **322**, 399.

PROCKOP, D.J. & UDENFRIEND, S.A. (1960). A specific method for the analysis of hydroxyproline in tissues and urine. *Anal. Biochem.*, **1**, 228.

ROSENSHEIN, N., BLAKE, D., MCINTYRE, P.A & 4 others (1978). The effect of volume on the distribution of substances instilled into the peritoneal cavity. *Gynecol. Oncol.*, **6**, 106.

DE ROY VAN ZUIDEWIJN, D.B.W., WOBBES, TH., HENDRIKS, TH., KLOMPMAKERS, A.A. & DE BOER, H.H.M. (1986). The effects of antineoplastic agents on the healing of small intestinal anastomoses in the rat. *Cancer*, **58**, 62.

DE ROY VAN ZUIDEWIJN, D.B.W., WOBBES, TH., HENDRIKS, TH., KLOMPMAKERS, A.A. & DE BOER, H.H.M. (1988). The effect of cisdichlorodiammineplatinum(II) on the healing of experimental intestinal anastomoses in the rat. *Surg. Res. Comm.*, **2**, 297.

SUGARBAKER, P.H., GIANOLA, F.J., SPEYER, J.C., WESLEY, R., BAROFSKY, I. & MEYERS, C.E. (1985). Prospective, randomized trial of intravenous versus intraperitoneal 5-fluorouracil in patients with advanced primary colon or rectal cancer. *Surgery*, **98**, 414.

WADLER, S., SCHWARTZ, E.L., GOLDMAN, M. & 4 others (1989). Fluorouracil and recombinant afla-2a-interferon: an active regimen against advanced colorectal carcinoma. *J. Clin. Oncol.*, **7**, 1769.

WIZNITZER, TH., ORDA, R., BAWNIK, J.B., RIPPIN, A., GRIFFEL, B. & HERZBERG, M. (1973). Mitomycin and the healing of intestinal anastomosis. *Arch. Surg.*, **106**, 314.